# Animal-based welfare indicators for dairy cows and their validity and practicality: a systematic review of the existing literature

**DOI:** 10.3389/fvets.2024.1429097

**Published:** 2024-07-11

**Authors:** Jenny Linstädt, Christa Thöne-Reineke, Roswitha Merle

**Affiliations:** ^1^Institute of Animal Welfare, Animal Behavior and Laboratory Animal Science, School of Veterinary Medicine, Freie Universität Berlin, Berlin, Germany; ^2^Institute of Veterinary Epidemiology and Biostatistics, School of Veterinary Medicine, Freie Universität Berlin, Berlin, Germany

**Keywords:** animal welfare, wellbeing, animal-based, dairy cow, welfare quality®, welfare indicators

## Abstract

Animal welfare is of increasing importance, with consumers preferring animal products made with ethical practices due to growing awareness. This shift highlights the need for reliable methods to evaluate welfare. This systematic review aims to assess the validity of current animal-based welfare indicators for dairy cows to aid farmers and agricultural professionals in evaluating and improving welfare amidst the lack of a clear legislative definition. The literature search spanned five databases: CAB Direct, PubMed, Scopus, Google Scholar and Livivo, covering publications in English and German from 2011 to 2021. Specific search terms were employed, and abstracts were screened for relevance. Publications were categorized based on exclusion criteria, with a final verification process conducted by three independent scientists. Research highlights correlations between welfare measures, farm characteristics and innovative indicators like hair cortisol concentration. Farming systems and housing methods significantly affect welfare, with pasture-based systems generally resulting in reduced lameness and improved behavior. Proper housing design and management practices are important, as they influence indicators like lameness and cleanliness. Heart rate variability and heart rate monitoring provide insights into dairy cow stress levels during milking and other stressors, making them valuable for welfare assessment. Biomarker research emphasizes the need to balance productivity and health in breeding strategies, as high milk production alone does not indicate good welfare. Behavioral studies and the human-animal relationship are key to understanding welfare. Precision Livestock Farming offers real-time assessment capabilities, although validation is needed. Stress physiology is complex, and while cortisol measurement methods are promising, further research is necessary. Assessment tools like the Animal Needs Index and routine herd data analysis are valuable for identifying welfare concerns. Key findings highlight the WQ® protocol’s effectiveness and versatility, the challenge of its time demands, and the DCF protocol’s promise for more practical and efficient welfare assessments. Commercial animal welfare audits should prioritize easily observable indicators and herd records due to logistical constraints in measuring biomarkers or heart rate variability. This focus on easily accessible indicators, such as body condition score, lameness, claw health, cleanliness, and somatic cell count allows effective welfare assessments, enabling prompt action to enhance wellbeing.

## 1 Introduction

The subject of animal welfare is becoming more and more important in society (1). Public awareness is growing and the consumer is interested in products of animal origin which were produced under animal welfare-compliant conditions (1). With Article 13 of the Treaty on the Functioning of the European Union, the term welfare was mentioned in a European law for the first time in 2009. The animal is referred to as a “sentient being” whose welfare requirements are taken into account in political decisions of the EU and the member states (2). This gives rise to the problem that although the term animal welfare has made it into the EU treaty (2) it is not defined what exactly it is, despite the fact that it has such a big social relevance. Animal welfare is a critical issue, as it reflects societal values and ethical considerations regarding the treatment of animals. The inclusion of animal welfare in the EU treaty (2) signifies its importance at a policy level. The lack of a clear definition complicates the implementation and enforcement of consistent welfare standards across member states. Dairy cows often face unique welfare challenges, including issues related to housing, feeding, milking procedures, and overall health management. Despite their significant role in agriculture and the economy, the absence of tailored regulations leaves a gap in ensuring their well-being. This gap highlights the necessity for the EU to develop and enforce specific guidelines that address the welfare needs of dairy cows.

In Germany, animal protection has been a legally binding constitutional norm since 2002, when it was enshrined in Article 20a of the Basic Law for the Federal Republic of Germany (3). Article 1 of the German Animal Protection Law states that the well-being of animals as fellow creatures must be guaranteed (4), without a definition of the term being offered in this context.

Furthermore, Article 11 of the German Animal Protection Law stipulates that livestock owners must carry out internal checks to ensure that the requirements of Article 2 are met (5). For this purpose, “suitable animal-related characteristics (animal welfare indicators)” shall be collected and evaluated (6). The farmer must carry out a self-assessment regarding a not clearly defined animal welfare, with suitable indicators, which are not listed.

For a long time, it was believed that when an animal performs well (e.g., milk production), it feels comfortable (7). In other words, an animal that does not perform well does not feel well. In the meantime, it has been proven that there is a connection between production diseases in dairy cows and breeding with focus only on performance (genetic overload). This means that individual risk of disease (e.g., peripartum diseases) also has a genetic component and therefore, improved management and husbandry conditions cannot prevent all cases of disease (7). In addition to valid animal welfare indicators, other actions are also required, such as rethinking breeding targets in livestock husbandry. Less diseases would also mean a better welfare.

Even if there is no official definition of animal welfare, there is a common ground for the definitions that were proposed by several groups of experts. For example, there is the concept of the “five freedoms” of the British Farm Animal Welfare Council, (today Farm Animal Welfare Committee, FAWC) (8). The concept was founded in 1979, and has since then been updated and revised several times. The five freedoms are as follows: “Freedom from hunger and thirst. Freedom from discomfort. Freedom from pain, injury or disease. Freedom from fear and distress. Freedom to express normal behaviors.” Webster also applied the concept of the five freedoms to livestock (9).

A description of the term also used by the O.I.E (World Organization of Animal Health) and created by Broom is that “the welfare of an individual is its state as regards its attempts to cope with its environment” (10).

In order to be able to measure animal welfare, animal welfare indicators come into play. In general, they can be divided into resource-based, management-based and animal-based indicators. Resource and management-based indicators assess animal welfare through the animal’s surrounding environment or housing and generally serve to prevent respective risks or threats. Animal-based indicators are results-oriented, evaluate animal welfare in the animal itself and thus provide a picture of the present status of the individual.

To evaluate welfare, tools are needed that can assess it in an objective, animal-based manner and are suitable for daily use. Many researchers have dedicated themselves to this task, so that there are now various evaluation systems, measurement protocols and other approaches.

One of the most popular assessment systems is the European Welfare Quality® Assessment Protocol, which contains an explanation of the procedure for evaluating the welfare of cattle (11). A working group of the German KTBL (Kuratorium für Technik und Bauwesen in der Landwirtschaft e.V.) also used the Welfare Quality® indicators to provide a guideline for the operational self-assessments (12).

In view of the large number of indicators, which are often difficult to measure, the question comes up, which indicators are most reliable and suitable for farmers daily self-assessment.

In this systematic review, the currently used indicators for the assessment of animal welfare in dairy cow farming are presented, discussed and assessed for their validity.

The focus is laid on animal-based indicators, because they can be successfully used in the evaluation of the welfare especially in the context of dairy cow farming in relation to laws, codes of practice, quality assurance schemes and management (13). Standardized valid animal-based welfare indicators could be able to improve the husbandry of dairy cows. The aim is also to provide farmers and other agricultural professions with assistance in evaluating animal welfare, as there is no clear definition at the legislative level.

## 2 Materials and methods

### 2.1 Databases and catalog of criteria

The literature research utilized five databases: CAB Direct, PubMed, and Scopus for English-language publications, and Google Scholar and Livivo for German-language literature. Publications in both German and English were considered. The publication years were restricted to the period from January 1, 2011 to October 20, 2021. In terms of content, the studies were limited to those geographically situated in Europe. Dairy cows were identified as the sole relevant livestock group for inclusion in the systematic review.

### 2.2 Search terms

Due to variations in the operational and selection elements among the five databases employed, the search methodologies differed as follows: in CAB Direct and Scopus, descriptors were searched within the abstracts. In PubMed, descriptors were searched within both the titles and abstracts. In Google Scholar and Livivo, there were no restrictions; hence, the descriptors could appear anywhere within the full text. Additionally, citations and patents were excluded from the search in Google Scholar.

For the German-language searches on November 7, 2016 and October 20, 2021, the following terms and combinations were chosen in Livivo:

Tierwohl Milch*.

Tierwohl Rind*.

Tierwohl Kuh.

Wohlbefinden Milch*.

Wohlbefinden Rind*.

Wohlbefinden Kuh.

Tiergerecht* Milch*.

Tiergerecht* Rind*.

Tiergerecht* Kuh.

Note: Replacing the search term “cow” with “cows” returned identical results in Livivo, so the search was limited to the descriptor cow.

The German-language search on December 14, 2016 and November 5, 2021 in Google Scholar was carried out with the following terms and combinations:

Tierwohl.

+ MilchkuhORMilchküheORMilchrinderORKuhORKüheORRindORRinder.

- SchafORZiegeORKalbORKälberORGeflügelORHuhnORHühnerORPuteORSchwein.

Wohlbefinden.

+ MilchkuhORMilchküheORMilchrinderORKuhORKüheORRindORRinder.

- SchafORZiegeORKalbORKälberORGeflügelORHuhnORHühnerORPuteORSchwein.

- FerkelORMannORFrauORKindORMusikORReligion.

Tiergerecht.

+ MilchkuhORMilchküheORMilchrinderORKuhORKüheORRindORRinder.

- SchafORZiegeORKalbORKälberORGeflügelORHuhnORHühnerORPuteORSchwein.

Tiergerechtheit.

+ MilchkuhORMilchküheORMilchrinderORKuhORKüheORRindORRinder.

- SchafORZiegeORKalbORKälberORGeflügelORHuhnORHühnerORPuteORSchwein.

The English-language search in CAB Direct, PubMed and Scopus on August 23, 2016 was carried out with the following terms and combinations, with German publications also being permitted:

dairyORcow*ANDwelfareNOTgoatNOTsheep.

dairyORcow*ANDwell-beingNOTgoatNOTsheep.

dairyORcow*ANDwellbeingNOTgoatNOTsheep.

This search was repeated on October 7, 2021, in Pubmed, and on October, 21, in CAB direct.

### 2.3 Abstract-screening and grouping

In the initial phase, the results of the database searches were categorized based on publication type. Simultaneously, a software-assisted cleanup using Citavi Version 5 was conducted to remove duplicates from the result list. Initially, the database entries totaled 5,119, which were subsequently reduced to 3,491 after the removal of duplicates. Further manual sorting eliminated additional duplicates, resulting in a final count of 2,818 database entries.

Subsequently, these entries or publications were grouped and, where necessary, their bibliographic information was completed. Initial classification of the publications included the following groups: “wrong species,” “wrong topic,” “outside Europe,” “uncertain relevance,” and “potentially relevant”.

Furthermore, a separate category labeled “completely irrelevant” was established, into which certain database entries were placed due to inaccuracies in the search algorithms and links to literature.

Additionally, entries pertaining to collective works and conference proceedings, which often serve as mere placeholders for individual titles, underwent cleanup. Some collective works required identification and linkage to existing individual titles, while others necessitated the creation of artificial entries to establish clear bibliographical associations. Ultimately, these placeholders were removed from the remaining publications, without altering the original number of hits retrieved from the database query.

All titles were systematically categorized based on the exclusion criteria. The title of each publication, along with its abstract when available, was thoroughly reviewed. In cases where essential information was missing, references were made to the full text.

The categorization process followed a hierarchical approach. For instance, if a publication described the wrong species (e.g., pig instead of dairy cow), the topic and study location became irrelevant. Incorrect life stages, such as calves, were also sorted out. Priority was given to species, followed by subject matter, and then study location.

Publications primarily addressing dairy cow owners or producers of dairy products and their perspectives on animal welfare were classified under the “wrong topic” category. Conversely, publications focusing on farmers’ attitudes and assessments of animal welfare in general, without specific emphasis on dairy cows or covering other livestock species, were considered “wrong species”.

Any database entries not related to animal welfare, well-being, emotions, or behavior specifically in dairy cows were excluded as “wrong topic”.

Furthermore, all ambiguous and potentially relevant publications were classified into original studies (peer-reviewed), reviews, or knowledge transfer (book chapters, guidelines, and other forms of “gray literature”). The subsequent analysis focused on original studies within these two categories.

### 2.4 Verification

**Figure 1:**
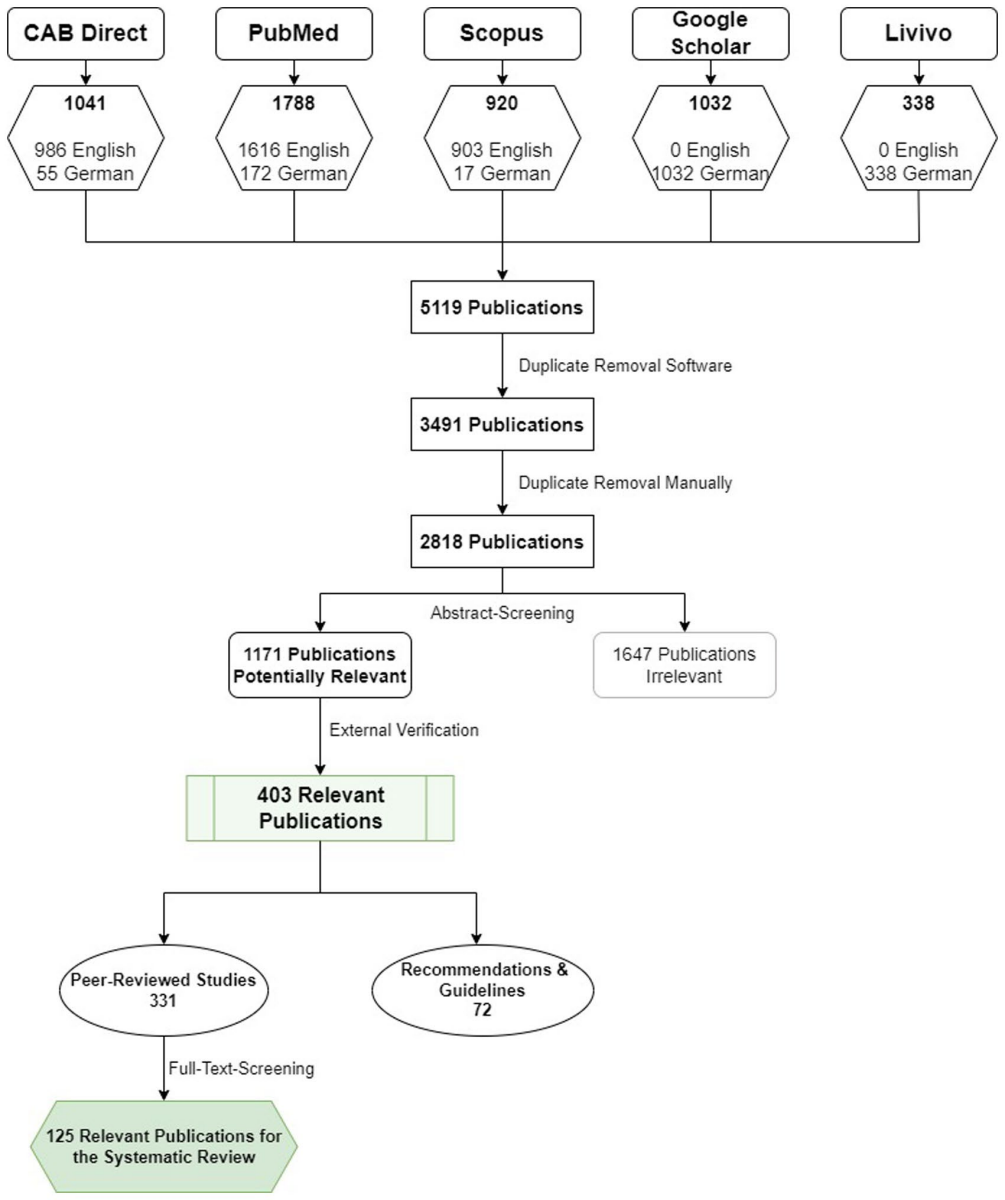
Selection process of research papers.

The assessment of the potentially relevant publications as ultimately relevant or not was carried out objectively by three scientists. 125 publications were verified and clearly assigned at this point. The verification process is illustrated in the form of a flowchart in Figure 1.

### 2.5 Risk of bias

The risk of bias was reduced to a minimum through the systematic approach and the creation of uniform search criteria for the literature search. In addition, the risk was reduced by the fact that 3 independent scientists evaluated using the same inclusion and exclusion criteria which were previously determined.

Quality assurance is ensured by the fact that all included studies underwent peer review and were additionally evaluated according to the “Standard Quality Assessment Criteria for Evaluating Primary Research Papers from a Variety of Fields” (14). For this purpose, the “Checklist for assessing the quality of quantitative studies” was utilized, and all papers were assessed based on the following criteria:

- Question/objective sufficiently described?
- Study design evident and appropriate?
- Method of subject/comparison group selection or source of information/input variables described and appropriate?
- Subject (and comparison group, if applicable) characteristics sufficiently described?
- Outcome and (if applicable) exposure measure(s) well defined and robust to measurement/misclassification bias? Means of assessment reported?
- Sample size appropriate?
- Analytic methods described/justified and appropriate?
- Some estimate of variance is reported for the main results?
- Controlled for confounding?
- Results reported in sufficient detail?
- Conclusions supported by the results?

Since all papers had already been confirmed as thematically suitable for the systematic review by the criteria mentioned beforehand, none of the papers were excluded, even if they received a low score. The quality assessment was not intended for further exclusion, but rather for evaluating the quality of the studies. A lower score can also be explained by a different format of the respective paper and does not necessarily mean that the quality of the paper is insufficient.

Scores of 2 were assigned for “Yes,” 1 for “Partial,” and 0 for “No.” If nothing applied, “N/A” could be used for some of the criteria. The scoring was conducted by Author 1 and Author 3.

**Figure 2:**
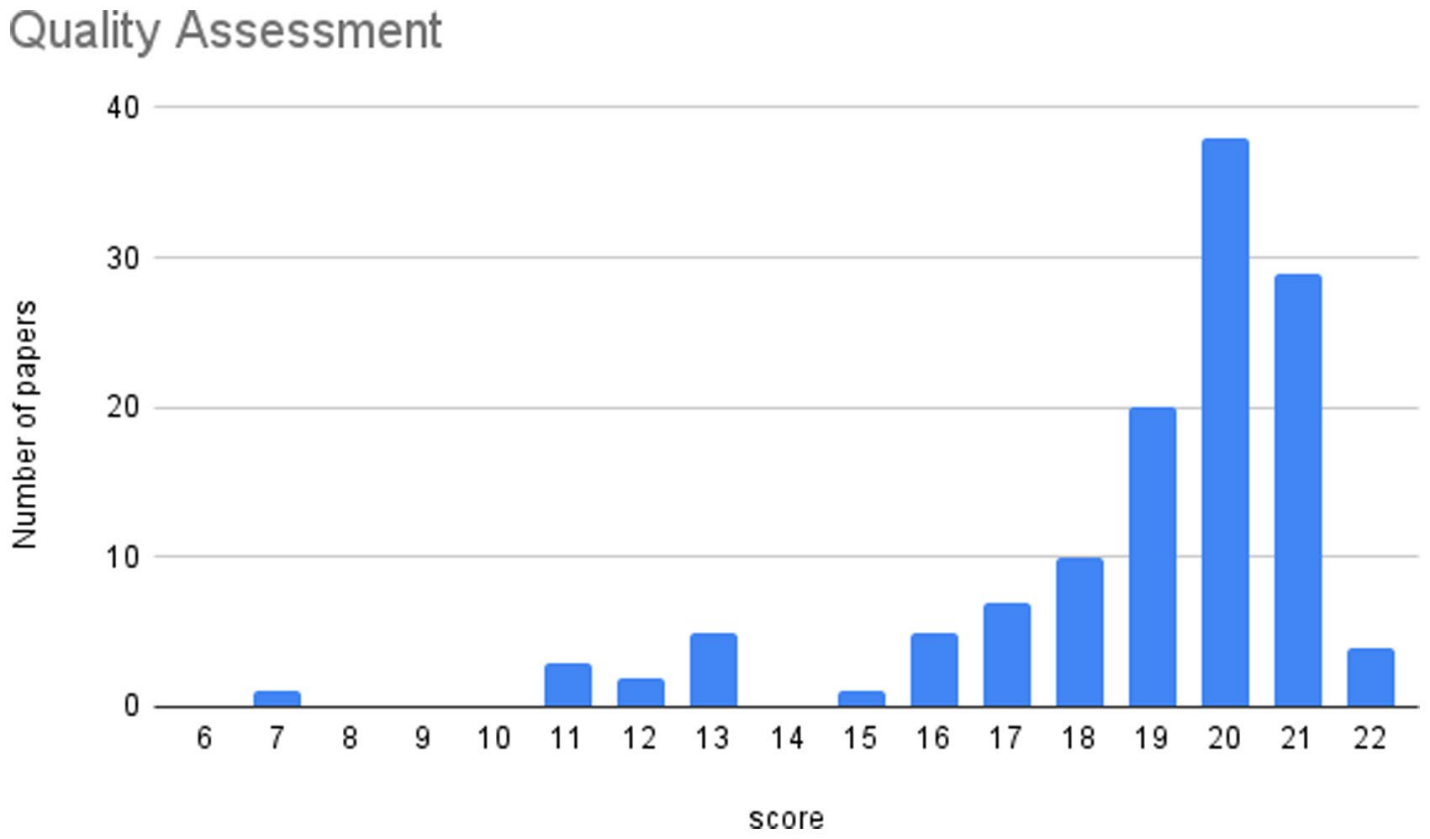
Quality assessment.

In the systematic review, papers numbered 15 to 139 in the list of citations were included. Among these, the highest attainable score is 22, which has been achieved by 4 papers. Notably, 73% of the papers scored 19 or higher, indicating a generally high level of quality across the included studies. Conversely, a small proportion, specifically 4.8% of the papers, scored 12 or lower. It’s worth noting that the paper with the lowest score of 7 is categorized as a research reflection. This lower score may be attributed to the fact that the checklist questions may not be entirely suitable for evaluating this particular type of text. This also applies to one paper, which scored 11 (ranking 49 in the list of citations), and another paper which scored 12 (ranking 53 in the list of citations), as they are reviews. Additionally, Paper 44 in the citations list also scored 11. This could be attributed to the study’s described inconclusive correlations, which can result in a lower score. The systematic review’s inclusion of a broad range of studies, with careful consideration of their quality and relevance, provides a comprehensive and reliable synthesis of the available research. This thorough approach ensures that the conclusions drawn from the review are well-founded and reflective of the overall evidence base. The distribution of the results of the quality assessment is shown in Figure 2.

## 3 Results

**Figure 3:**
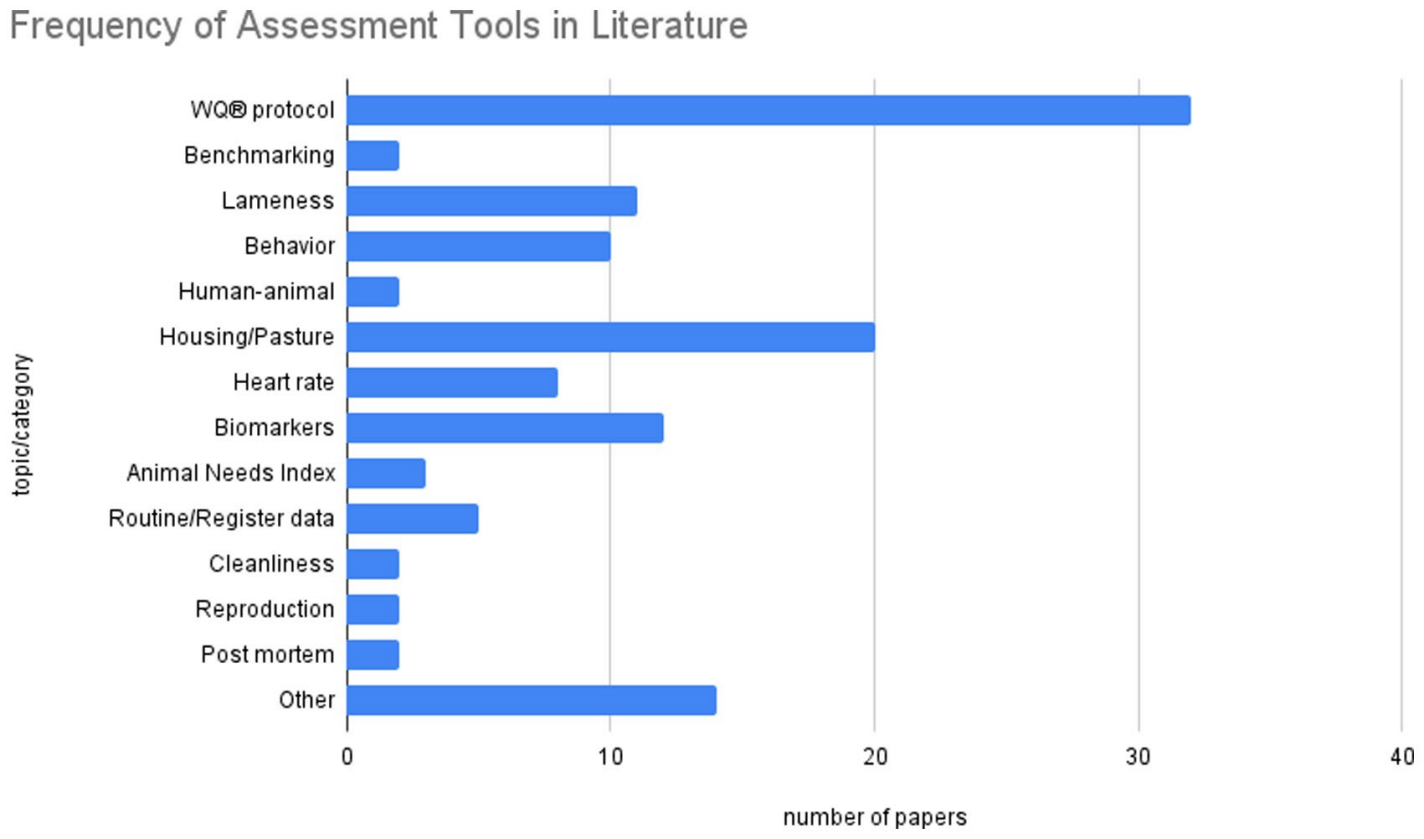
Frequency of assessment tools in literature.

The systematic review yielded a comprehensive overview of studies across various topics related to welfare indicators. The frequency of each topic/category within the literature is quantified as shown in the following bar chart. The frequency of each topic/category within the literature is quantified as shown in Figure 3.

Out of the selected studies, a significant amount refers to the WQ® protocol, with 32 papers dedicated to this topic. The second most common category is “Housing/Pasture,” with 20 papers. The topic of biomarkers is also frequently addressed, appearing in 12 papers. “Lameness” with 11 papers and “Behavior” with 10 papers are also significant topics in the measurement of welfare. The category “Other” consists of papers that could not be assigned to any of the specific categories and for which there was only one paper, making it impossible to form additional categories. The “Animal Needs Index” was examined and applied in 3 papers. Less frequently discussed topics include “Human-Animal Relationship,” “Benchmarking,” “Cleanliness,” “Reproduction,” and “Post-mortem,” each addressed in 2 papers. It is worth noting that “Cleanliness” is a component of the WQ® protocol and therefore is applied more frequently, though not as a standalone indicator, but in combination with other indicators. The following sections present the results of the research in detail.

### 3.1 Welfare quality® protocol

One of the most important assessment approaches in the EU is the Welfare Quality® protocol. Although there is no gold standard for evaluating animal welfare, the WQ® protocol is very often referred to. It consists of 30, mostly animal-based welfare indicators and is divided into four principles: good housing, good feeding, good health, and appropriate behavior (11).

The WQ® protocol is widely accepted and has been validated in several studies (15–19) and proven to be useful. Gieseke et al. (15) applied the WQ® protocol as part of a field study and statistically analyzed the data to evaluate the WQ® protocol. They could prove that the WQ® protocol offers good prerequisites for recognizing farm-specific risk factors and recording animal welfare at farm level (15).

The WQ® protocol has been successfully used in various studies (15–19) to measure animal welfare. For example, Coignard et al. (16) showed, that the overall health of dairy cows (130 farms were assessed) was moderate but ranged with the farming system. In a study from Macedonia in 2014 (17), it was disclosed that the most welfare concerns are ascertained in the WP Good Feeding and Good Housing. Another study, which investigated three dairy cow farms concerning the common health disorders, assessed the three farms as “acceptable,” which means that the provided welfare circumstances performed the minimum needs of animals (18). Several health problems were assessed which differed between the farms. One farm had more occurrences of skin injuries than the other two. Other detected problems were for example reproductive disorders and lameness (18). In another paper, the assumption that “monitoring of welfare could increase the profitability of dairy herds by improving indices of reproduction” was tested and the authors found remarkably positive correlations between welfare parameters, reproductive indices and milk production (19). In 2020, Bugueiro et al. studied 31 dairy herds and used the WQ® protocol to identify fields in which the surveyed herds should improve (20).

In an approach (21) on 34 Austrian dairy farms, the farms were assessed two times within 1 year. The farmers received a written report and were invited to apply improvement measures in husbandry and management. The result was an implementation rate of 57% of the recommendations, a notable refinement of udder health and cleanliness of teats, but no improvements in leg health (21). One point in which many experts seem to agree, is that the execution of the WQ® protocol is very time consuming (22–26) and takes a full day (6-8 h) to perform. This circumstance makes the application of the WQ® protocol expensive (22, 24) and resulted in various attempts to change or shorten the WQ® protocol without changing the accuracy of the measurement.

The Danish Cattle Federation (DCF) developed a simple welfare assessment protocol and compared it to the WQ® protocol (22). The new DCF protocol consists of 14 measures, 13 of which are animal based, and that takes 2 hours to apply. In an extended version, it reached a significant correlation with the WQ® protocol on all levels. Despite the overall high correlation, some specific areas showed only moderate correlation. For instance, “water provision” in the extended DCF protocol showed moderate correlation due to differences in how fat animals are considered. Similarly, for “positive behavior,” the DCF protocol uses avoidance distance as a measure, which does not fully capture the aspects included in the WQ® protocol, such as grooming and pasture access.

The extended DCF protocol’s use of simpler, animal-based measures, and fewer cows inspected (16% compared to WQ®‘s 38%), makes it more practical and time-efficient for routine farm use. However, this comes with the risk of false positives and negatives in welfare assessments. Despite this, the time saved and the practical focus on animal-based measures make the DCF protocol a viable alternative.

The DCF protocol uses relative percentiles based on the population, which will change as welfare levels change, unlike the absolute scores of the WQ® protocol. This makes the WQ® more suitable for cross-country comparisons and labeling, but the DCF’s simpler summarization method is more transparent and user-friendly.

The extended DCF protocol was developed specifically for Danish conditions and worked well for cattle in Denmark (22).

Another approach to reduce the time for assessment was to identify the so-called “iceberg-indicator,” which is believed to provide an overall assessment of welfare. The paper concludes that by only measuring one single, resource-based score, the absence of prolonged thirst (WQ® criteria score), the correct welfare classification can be obtained in 88% of the cases (23). The assessment time took 15 min.

A further attempt to reduce the assessment time was presented in a paper from van Eerdenburg et al.: to exchange the most time consuming parts of the WQ® protocol, like for example the behavioral observations, by environment-based measures and other modifications (25). It succeeded as a practical tool that takes 1,5 h to perform on a farm with 100 cows.

In the WQ® protocol, Qualitative Behavior Assessment (QBA) (11) is a method used to evaluate animal welfare based on observers’ subjective scoring of behavioral expressions such as posture, activity level, and facial expressions. It provides a holistic understanding of animal welfare on farms by considering the overall impression of behavior rather than focusing on specific behaviors or physiological parameters (11).

In 2012, a paper was published in which the authors also tried to solve the time problem by using QBA as stand-alone assessment approach to determine farms with limited welfare conditions before performing the whole WQ® protocol (26). No significant correlations could be detected, so the study does not recommend the use of QBA as a single measurement tool.

The next approach is also a reduction of the WQ® protocol, in which four different assessment methods were used: avoidance distance at the feeding rack; QBA; behavioral observations and clinical observations. The conclusion was that it is not recommended to leave out indicators of the WQ® protocol, but to use additional data or automated monitoring systems in terms of time-reduction (24).

Another method to make the WQ® protocol more user-friendly was presented by Tuyttens et al. (27): they used only a few key measures to assess the welfare of the animals, and combined them into a single welfare index (WI). The indicators were determined through expert surveys (lameness, leanness, mortality, hairless patches, lesions/swellings, somatic cell count). The simplified protocol turned out to be consistent with the opinions of experts and the time needed to carry out the assessment was reduced by a factor of 2–3. The authors recommended to include a disclaimer that outlines both positive and negative effects that may not be accurately detected by the current set of measures (27).

Van Eerdenburg et al. (28) developed a scoring system for free stall barns to observe the dairy cow comfort and examined the impact on the milk yield. They took animal-based parameters and environmental aspects and found that they needed significantly less time to apply the system compared to the WQ® protocol. A positive correlation between the used free stall parameters and milk yield was found (28).

Studies using databases and epidemiological approaches have explored various aspects of dairy cow welfare. Otten et al. (29) aimed to construct animal welfare indices (AWI) from data of 73 Danish dairy herds to compare and validate register- and resource data against animal-based data, concluding that on-farm animal welfare assessments with animal-based indicators were more reliable due to limited correlations between indices and predictive key indicators (29). In a study from 2015 (30), a national database was used in order to find dairy cow farms with insufficient animal welfare conditions. Out of this database, which contains registrations of cows and their deaths and movements, 14 million records were evaluated to discover and figure out 15 possible welfare indicators. An on-farm welfare assessment with the WQ® protocol was carried out on 24 farms for comparison. In conclusion, the two variables “proportion of on-farm deaths” and “calving-to-calving-interval” helped to identify farms with poor welfare (30).

Accurate welfare measurement was discussed by assessing different sampling strategies, showing that low-prevalence measures required more cows for accurate estimates (31). In a 2014 study (32), an epidemiological approach was used to investigate welfare issues in French dairy cows, identifying pain, bad health, and poor resting comfort as significant problems (32).

Kirchner et al. assessed 30 dairy farms (33) using the WQ® protocol, identifying weaknesses like injuries and discomfort in lying areas, and noting that organic and low-input systems can achieve good welfare results, though access to pasture did not always meet “Excellent” standards (33).

In their study, Wagner and colleagues (34) found that organic farms scored higher in all WQ® principles compared to conventional farms, but both showed room for improvement in “Good Health” (34).

A 2020 study examined the influence of cubicle traits on animal welfare, finding bedding type to be the most influential factor (35). Popescu et al. compared tie-stall and free-housing systems, finding that most evaluated farms had “unacceptable” welfare, with insufficient water supply being a major issue (36). The impact of the daily grazing time of cows on their welfare was determined (37) and the results showed that in farms where grazing was available, the welfare of cows improved from winter to summer. Positive effects cannot be ensured if the general management fails to meet the requirements of the cows (37).

In 2015, the replicability of QBA outcomes was examined, across three distinct observation periods throughout the day (early morning, late morning, early afternoon) (38). For certain farms, QBA results may differ considerably based on the time of day when the evaluation is conducted. As recommended in the WQ® protocol, using a standardized observation time facilitates observing the animals under similar conditions, thereby ensuring a high level of comparability (38).

In the following study, the reliability of QBA for assessing the welfare of dairy cattle was examined by analyzing videos and comparing the observations of experienced and inexperienced observers (39). The results showed that the agreement between different observers varied from slight too high for individual QBA descriptors and from slight to moderate for QBA scores. Additionally, there were differences in the values assigned by experienced and inexperienced observers for half of the descriptors and the QBA score (39).

In the following study, the authors asked whether people who are trained in using the WQ® protocol for dairy cattle have the same opinions as the scores calculated by the WQ® protocol (40). Their findings revealed that certain measures that were deemed less important by experts had a greater impact on the overall welfare categorization of the WQ® protocol. Conversely, measures that were considered highly important by experts had a lower effect on the overall welfare categorization. Specifically, measures related to drinkers had a significant impact on the welfare categorization, while these related to lameness and mortality had a lower effect (40).

In a paper from 2020 (41), it was also considered whether the WQ® protocol could be implemented with sensor technologies. It was stated how current precision livestock farming technologies have the capability to evaluate the majority of WQ® indicators. Although certain welfare indicators may not be suitable for sensoring technologies, alternative measures that evaluate the same welfare criteria could be used as a substitute. It is expected that in the future, there will be an increase in the availability of objective and continuous data provided by precision livestock farming technologies (41).

Four scientists analyzed three widely recognized systems (WQ®, FARM and The Code of Welfare), highlighting their strengths and weaknesses (42). Expanding the scope of environmental measurements could potentially enhance the ability of WQ® to identify the environmental factors that impact the welfare outcomes observed in cows (42). De Vries et al. focused on the key welfare measures influencing WQ® classification in Dutch dairy herds, suggesting a need to reconsider the role of expert opinion and the algorithmic operator to improve classification (43).

A survey on 131 French dairy farms using the WQ® protocol identified major welfare issues, emphasizing the importance of farm-specific characteristics in welfare plans and continuous improvement in health, behavior, and feeding (32).

As an innovative approach, hair cortisol concentration, reflecting long-term stress, has emerged as a potential indicator (44). However, the correlation between Welfare Quality® scores and pooled hair cortisol concentrations remains inconclusive, necessitating further research with larger sample sizes and standardized protocols, according to a study by Vesel et al. Various factors, including sampling time, cow characteristics, and environmental conditions, influence hair cortisol levels, demanding meticulous attention in future studies (44).

Bergschmidt et al. (45) had the idea of including an approach in the EU’s Rural Development Program, where the payment for the farmers is depending on the results. So far, the payments have been based on actions like a welfare friendly housing or management, neglecting welfare outcomes. In a multi-step process, which involved a literature review, a written Delphi survey with scientists, a group discussion with stakeholders („practioner workshop“) and on-farm trial, 10 indicators were selected. An assessment of the WQ® protocol has also taken place. The project indicators and the WQ® protocol exhibited a limited level of coherence in their comparison.

It is said that dairy cattle support measures can cover all aspects of animal welfare, including health, behavior and emotions, with the help of action-oriented requirements and outcome-oriented indicators in combination (45).

In 2017, a paper was published with the aim to demonstrate the necessity of adjusting some elements of the WQ® protocol in tropical regions, so it would be even more useful (46).

### 3.2 Benchmarking

Benchmarking plays a role in evaluating and enhancing the performance of dairy cow farms by comparing their practices and outcomes to industry standards and best practices. In 2018, a welfare assessment protocol has been created specifically for small-scale dairy cattle farms that practice vertical transhumance (47). It is based on the WQ® protocol. In a sample of 67 farms, 18 nonbehavioral animal-based measures were evaluated before, during, and after the mountain pasture period. The purpose of this study was to present field data from the transhumant system and to identify intolerable welfare affairs. To contribute to the discourse on achievable welfare results for the two husbandry conditions that define a transhumant system, a benchmarking exercise was conducted. The aim was to determine the comparative limits (thresholds for the lowest quartile) for each animal-based measure. The results show that a significant number of cows (65%) had bald spots before being put out to pasture. When cattle were housed indoors, there was a notable prevalence of 80% of them being found to be dirty. Additionally, more than 13% of the cows were identified as very thin (47).

Trillo et al. successfully used a benchmarking process in 73 dairy farms in Spain to detect negative points and improvable aspects. Animal-based indicators were assessed, which led to the outcome that hock lesions and lameness are common problems, also like a suboptimal Body Condition Score (BCS) (48).

### 3.3 Lameness

A review from 2011 emphasizes the behavioral implications of lameness, exploring the interplay among locomotion scores, lying patterns, and milking parlor positioning (49). The presence of clinical lameness not only induces chronic stress but also has an effect on reproductive hormones and sexual behavior. Hoof diseases, contributing to pain, further jeopardize overall welfare. Enhancing comfort, especially in lying areas, emerges as a key strategy to mitigate lameness and promote holistic health, with straw bedding demonstrating notable advantages. Nonetheless, it’s crucial to recognize that relying solely on measuring lying behavior may not provide an accurate gage of lameness severity (49).

O’Connor et al. defined the quality of mobility by investigating the connections among particular mobility scores, claw disorders, BCS and cow parity (50). Data was gathered for 6,927 cows from 52 dairy herds. These data encompassed mobility scores („0 = optimal mobility; 1, 2, or 3 = increasing severities of suboptimal mobility “), the type of claw disorders, the BCS, and the parity of each cow. Based on the results, it’s apparent that a correlation exists between mobility scores and claw disorders among dairy cows in pasture-based systems. Moreover, the research establishes links between BCS, cow parity, and mobility scores. Notably, claw disorders with severity scores <2 were tied to an elevated risk of developing mobility score 3 in contrast to score 0. This emphasizes the effectiveness of mobility scoring in identifying cows with mild claw disorders at an earlier stage (50). In another study, they correlated mobility scores with reproductive performance and production measurements (51). Their research indicates that poor mobility in dairy cows during spring-calving in pasture-based systems is linked to reduced production (lower milk, fat, and protein yields, along with higher somatic cell count (SCC)) and compromised reproductive performance (longer calving intervals) (51).

To further investigate “lameness,” it is discussed how it impacts cow welfare through a comparison of regular and irregular gaits, including the utilization of Locomotion Scoring (LS) systems for detecting lameness (52). Implementing LS to identify lame cows demands clear gait feature criteria to enhance result consistency. However, practical use is constrained by the need for proper farm facilities to guarantee precise outcomes (52).

A review from 2012 analyzes how lameness affects the behavior of intensively managed dairy cows (53). Lameness influences social rank, with affected cows losing positions in the food trail and milking order. This impacts productivity and survival (53).

Concerning to Weigele et al., mildly lame cows show distinct behavior changes from nonlame ones, affecting lying, activity and feeding patterns (54). These alterations, like reduced movement and extended lying, impact physical well-being and energy balance, potentially leading to more health issues and shorter lifespans. Limited mobility may also weaken resilience and social behaviors. This underscores lameness’ early and significant impact on animal welfare for moderately lame dairy cows in open housing (54).

Another study also explored the connection between lameness and changes in feeding behavior (55). Using gait scoring and monitoring feeding behavior, intake, milk yield, and weight, the researchers found that cows with more severe lameness spent less time feeding daily. They used electronic feeding troughs and automatic milking systems (AMS) for the measurements. They also found an interaction between lameness score and parity, with severely lame first-calving cows feeding the least. Profoundly lame cows ate faster but had lower body weights in comparison (55).

Another approach was also carried out using AMS. These systems use technology to automate milking procedures, offering advantages in terms of efficiency, cow welfare and data collection.

Three studies from 2013 showed that lameness in high-yield cows within an AMS affects feeding, rumination, and AMS visits (56). This has negative implications for farm profitability and cow welfare. Further research is necessary to optimize AMS technologies for health monitoring (56).

In recent years, the utilization of technology in the dairy industry has extended beyond milking processes to encompass the area of cow welfare. Automatic lameness detection systems have emerged as a helpful tool. The aim of the next study was to develop and validate a model for detecting lameness based on daily activity data (57). Automated lameness detection can rely on daily fluctuations in animal behavior. Activity sensors that monitor parameters like lying time and bouts were employed to record behavior of every cow per day. The lameness detection model showed consistent results between development and validation sets. Sensitivity reached 85.5%, making the model practical, though 88.8% specificity might need enhancement. According to the authors of the study, behavioral shifts as indicators of lameness hold potential (57).

Nechanitzky et al. (58) also assessed indicators for automated lameness detection in cubicle barns. They involved 32 lame cows with one hind limb claw horn lesion and 44 healthy nonlame cows. Nighttime lying and standing behavior were recorded by accelerometers, hind limb weight distribution by weighing platforms, feeding behavior by nose band sensors, and heart activity by Polar devices (58). Locomotion score correlated positively with lying time and weight difference, negatively with limb weight ratio and deviation. The best predictor of lameness included weight deviation and lying time. They concluded that weighing platform data, with or without lying time, are valuable for identifying claw horn lesions in one hind limb lameness. Feeding behavior and HRV variables have minor relevance (58).

In a review by Leliveld et al. (59), the need to integrate various welfare indicators to create a comprehensive assessment of dairy cow welfare on farms is highlighted. The focus is on developing an integrated automatic system to detect issues like lameness, heat stress, and pain. The study identifies common indicators, such as reduced feed intake, suitable for detecting overall reduced welfare, and specialist indicators, like increased respiratory rate for heat stress. Combining these indicators offers the potential for an early warning system in addressing welfare problems (59).

### 3.4 Behavior

Dairy cow behavior serves as a window into the dynamics between animals and their environment within modern farming systems. By studying how cows interact, move, and respond to various stimuli, valuable insights into their well-being, health, and overall performance can be gained. In the context of three open cowsheds, a study from 2012 investigated the impact of the potentially stressful waiting area of a milking parlor on dairy cows’ behavior and welfare (60). The research encompassed 3,522 individual cow observations. Waiting times, varying based on factors like group size and milking parlor capacity, reached up to 1 h, 42 min, and 22 s. Cowsheds I and II saw only around one-third of cows ruminating in the waiting area, while Cowshed III, with the smallest feeding group, shortest waiting time, and most space per cow, observed up to 52% of cows ruminating. Extended waiting times curtailed normal behavior opportunities for cows, indicating compromised welfare (60).

The study from Hedlund and Løvlie showed that links between personality traits and production are behavior-specific, influenced by milk measurements and breed (61). A common trend indicated that behaviors associated with cow nervousness were linked to reduced milk production. This alignment with resource allocation theory suggests negative correlations (61).

Two studies investigated the impact of omitting scheduled milking on cow comfort indicators (62). Decreased lying time, increased mammary pressure, and higher milk leakage resulted from reducing milking frequency from twice to once daily, either temporarily during lactation or weekly. Rapid behavioral and physiological adaptation, restoring parameters to pre-omission levels, were observed in both studies. Hence, immediate cow comfort wasn’t significantly affected by transitioning to once-daily milking or skipping a weekly session. It is said that more research is needed to assess long-term effects on cow welfare (62).

There are several papers in which automated devices were used to collect data about dairy cow behavior. A study from Italy compared behavioral indices from diverse scan-sampling frequencies, focusing on lying and standing behaviors (63). Video recording of 69 cows’ behaviors over a week, with Temperature Humidity Index (THI) logged every 15 min, unveiled insights. Results from hourly interpretations of lying, standing, and feeding behaviors, especially between daily milkings and evening hours, showed strong correlations to 10, 20, and 30-min scans. Night hours had limited impact. Farm management was significantly linked to cows’ activity 1–2 h post-milking. Video systems proved effective for cow activity analysis (63).

Validating the AfiTagII device’s accuracy in measuring lying behavior was the goal of another study (64). The device, attached to cows’ hind legs, showed high correlation with direct observations of lying time. Frequency of lying bouts had a positive predictive value of 0.96 for lactating cows on slatted floors and 0.85 for dry cows on deep bedding, compared to direct observations. The AfiTagII accurately estimates lying behavior in Danish Holstein and Jersey cows, regardless of bedding material or breed. However, skin lesions developed in some monitored cows, highlighting the need for device improvements (64).

Another approach used accelerometers to classify cow behaviors (65). Combining neck and leg data achieved precise (80–99%) and sensitive (87–99%) behavior classification. Neck accelerometers performed better for feeding (92% precision, 97% sensitivity) than leg ones (80% precision, 88% sensitivity). Classification accuracy depends on sensor position, sampling rates, and axes (65).

Using automated sensors, Ramón-Moragues et al. (66) tracked behaviors of 40 cows under varying heat stress conditions. The aim was to identify heat stress-induced behavior changes. All behaviors were affected by environmental conditions, and the cows adapted by modifying their actions. The sensors proved valuable in capturing these adaptations, potentially paving the way for an early warning system based on behavioral shifts. Heat stress influenced behaviors like breathing, eating, resting, and activity. As the Temperature-Humidity Index increased, feeding, rumination, and resting times decreased, while panting and activity increased. Behavior patterns also changed during cooler times of the day (66).

Having explored technical devices to examine cow’s behavior, the focus shifts to the significance of grooming substrates in promoting welfare. Providing grooming materials is said to address the natural behaviors of cows and shall contribute to stress reduction. McConnachie et al. (67) studied dairy cows’ motivation for an automated mechanical brush. Cows were taught to unlatch a weighted barrier for access to fresh feed (positive control), a mechanical brush, or an unoccupied area (negative control). They gaged the weight cows would push for each resource. Cows demonstrated comparable motivation for fresh feed and the brush, despite varying data collection approaches, with lower motivation observed for the empty space (67).

In their invited review, Tucker et al. (68) delve into the factors that influence cows’ motivation to lie down and explore the consequences for their health and overall biological function when this behavior is impeded. The research sheds light on a range of environmental and cow-based factors that impact lying time, underscoring the significance of offering appropriate lying areas on farms to enhance animal welfare. Although increased lying times typically signify cow comfort, exceptions may arise due to factors such as disease or specific behaviors. When evaluating animal welfare based on lying time measures, careful consideration of individual contexts is essential (68).

In a thematically related study, Vanhoudt et al. (69) aimed to assess the variability of the indices “cow rumination” and “lying behavior” in a herd with an automatic milking system under stable husbandry conditions. Over 28 days, standing index, cud chewing index, and rumination index were monitored. The lowest variation occurred between 240 and 270 min after cubicle bedding refreshment for standing and rumination indices, and between 120 and 150 min for the cud chewing index. Despite consistent practices, there was still significant variation, suggesting the need for repeated measurements over consecutive days for reliability (69).

### 3.5 Human-animal relationship related indicators

Exploring the human-animal relationship within the dairy cow industry is important and has influence on choices for animal welfare and ethical considerations.

In alpine traditional husbandry systems, Battini et al. (70) examined the durability of Avoidance Distance (AD) tests as a means to evaluate dairy cow-human interactions over the long term. However, in this study, the consistency of AD varied throughout the year due to the distinctive nature of these traditional alpine systems. After the grazing period, the avoidance distance tends to be higher. This is attributed to significant shifts in the quality and quantity of human-animal relationship (70). Haskell et al. (71) posed the question: “Is the response to humans consistent over productive life in dairy cows?“. Unpleasant interactions can affect welfare and productivity, prompting the inclusion of fear-of-humans tests in welfare assessments. However, practicality limits testing all animals on large farms. For sub-sampling, age impacts responses, shown by testing 114 Holstein cows across various productive stages. Cows became more approachable and less nervous with age until mid-1st lactation. Consistent rankings within groups across stages were observed (71).

### 3.6 Housing/pasture

The choice between housing and pasture systems for dairy cows is an important decision in modern agricultural practices. Striking the right balance between confined housing and access to open pasture directly influences animal welfare and milk production. There are a lot of different approaches to this topic.

A review published in 2016 compared the welfare of dairy cows in continuous housing and pasture-based systems (72). Despite advocating for continuous housing, pasture-based systems generally offer better welfare and health. Pasture-based cows have less lameness, hoof issues, lesions, and diseases compared to continuous housing of cows. Pasture access improves behavior, lying/resting times, and reduces aggression. Cows prefer pasture over indoor housing, especially at night. Yet, challenges include a negative energy balance and weather exposure in pasture systems. In conclusion, incorporating pasture access brings significant animal welfare benefits to dairy production (72).

The authors of the next paper also wanted to find out which form of husbandry would be better: pasture-based vs. confinement-based management (73). They used a three-sphere framework – biological functioning, natural behavior, and affective states – to assess wellbeing. Pasture-based cows have lower risks of various health issues, including mastitis, claw lesions, and lameness, but higher risks of internal parasitism and malnutrition (73). They also exhibit more normal behavior patterns. However, pasture-based cows might face challenges such as extended periods away from pasture and climate-related stress. Hybrid systems can alleviate negative effects by combining confinement and pasture elements. Ultimately, an optimal system allows cows some choice between environments, with effective management being key to ensuring good welfare (73).

A study from 2012 aimed to assess the impact of summer grazing on the welfare of dairy cows in contemporary cubicle loose-housing systems (74). The within-herd comparison of 41 Danish dairy herds revealed that summer grazing significantly improved overall cow welfare compared to full-time winter housing. The welfare index (WI) was lower in summer, indicating better welfare, with improvements in integument condition, claw conformation, and better access to water and food. The study suggested that many daily grazing hours were more beneficial than fewer hours for dairy herd welfare, emphasizing the positive effects of summer grazing on cow well-being (74).

In temperate regions, where cows graze on pastures, limited access to grass could lead to nutritional deficits, possibly affecting their wellbeing (75). A study from 2015 examined how daily herbage allowance (DHA) affects dairy cow behavior, locomotion, and hoof health. Cows were assigned to eight treatments based on experimental duration (2 or 6 weeks) and DHA levels (60, 80, 100%, or 120% of intake capacity). While daily lying time remained consistent, DHA influenced the duration of lying bouts, with higher DHA linked to shorter bouts. No significant effects were found on locomotion or hoof health. Although altered behavior and locomotion may not directly imply impaired welfare, they could indicate hunger or potential hoof issues. This research offers valuable insights for further exploration into hunger-satiety status and hoof health, aiding in improved dairy cow management in intensified pasture-based systems (75).

Exploring welfare markers in dairy cows across distinct loose housing arrangements (deep litter vs. cubicle barns) using recycled manure solids as bedding material, the research of Molina et al. (76) uncovered vulnerabilities in feeding and health indicators within both housing types. The comprehensive welfare evaluation, considering feeding, shelter, and health metrics, revealed no distinguishable variations between farms implementing deep litter or cubicle barns. This implies the potential to attain favorable welfare circumstances regardless of the selected housing (76).

In the following examination, the welfare of dairy cows in Ireland’s spring-calving, pasture-based systems during grazing and housing periods was explored (77). Seven welfare indicators were analyzed on 82 farms. Lameness, BCS, and tail injuries were issues, but ocular health was positive. Nasal discharge was lower during housing. Cows showed avoidance behavior in response to humans. Opportunities for improvement were identified, and top farms set benchmarks: 0 to 5% clinical lameness, 0 to 12% cows with BCS outside range, 0 to 27% ocular discharge, 2 to 16% nasal discharge, 0% tail injuries, 0 to 14% integument alterations, and 4 to 74% avoidance distance of >1 m. These targets can enhance cow welfare in spring-calving pasture-based systems (77).

In another approach, lying and walking activity of 29 cows was monitored using pedometers (78). Over 18 days, observations were conducted during pasture access and indoor housing periods. Pasture-grazing cows exhibited lengthier lying periods with fewer bouts, suggesting enhanced comfort and reduced restlessness. Outdoors, lying behavior was more synchronized, with the majority of the herd lying down simultaneously (78).

Heinz et al. (79) aimed to explore the relationship between claw health in dairy herds and various herd parameters, focusing on housing conditions. Data from four large dairy farms in northeast Germany, covering 18,119 observations of 3,690 cows, indicated that effective herd health management significantly improved claw health. The analysis revealed that farms with solid concrete flooring and deep-bedded cubicles had lower risks of claw disorders compared to those with concrete slatted floors and high cubicles. The frequency of functional hoof trimming, carried out two or three times per year, positively influenced claw health. The study emphasized the importance of optimal housing conditions and meticulous herd management in reducing the risk of claw lesions in dairy cows (79).

Twenty-nine Holstein-Friesian dairy cows experienced 18 days of overnight pasture access and 18 days of continuous indoor housing in a crossover experiment (80). Cattle learned to move towards a bucket location that offered a reward, while avoiding an unrewarded one. They were then presented with intermediate “probe” buckets. Probing these buckets indicated optimism in judgment, reflecting positive emotions. Although probe bucket approach latency did not differ between treatments, cows took longer to approach the known rewarded bucket with pasture access than indoor housing. These results suggest that pasture access in cattle reduces anticipation of known rewards compared to indoor housing, potentially leading to more positive emotional states in pasture environments (80).

Popescu et al. (81) compared the welfare of dairy cows in loose housing vs. tie-stall systems and test the hypothesis that loose housing leads to better welfare. Altogether, 2,624 milking cows on 60 commercial farms were evaluated using measures from the WQ® protocol. Notable differences were observed in most parameters and welfare principles, favoring the loose system. Tie-stall farms were mainly acceptable, while most loose housing farms were categorized as enhanced (81).

Another study compared the welfare of dairy cattle in different housing systems across six farms (82). Results indicated that the loose housing system had advantages in terms of cow comfort and health. The tie housing system showed higher indicator values of discomfort and management gaps related to hygiene and disease (82).

By comparing welfare between two tie-stall housing systems: those with and without outdoor exercise, significant differences were observed, indicating exercise positively impacts tethered cows’ welfare. Farms allowing outdoor access had better welfare scores than those with permanent tethering, except for hunger and social behaviors (83).

In a paper from 2014, the authors assessed human-animal relationships (HAR) in dairy farms with tie stalls and loose housing (84). Observations and tests on 424 cows showed that tethered cows tend to be calmer, trusting, and less fearful of humans compared to loose-housed cows (84).

The influence of different bedding materials on well-being was examined in a study from 2014 (85). In farms utilizing straw bedding, dairy cows exhibited cleaner flanks, upper hind legs, tails, and udders compared to those with sawdust bedding. A greater proportion of cows in straw bedded farms had hairless patches on their tarsus area than in sawdust-bedded farms. The assessment of overall cow welfare across the visited farms resulted in either enhanced or acceptable ratings. More farms using sawdust were classified as enhanced, while those using straw were categorized as acceptable for cow welfare (85).

De Vries et al. (86) wanted to discover and compare the effects of housing and management factors on the occurrence of lameness, lesions or swellings, dirty hindquarters, and displacements in dairy cows housed in free-stall systems. The research identified 15 significant factors related to these indicators of cattle welfare. Notably, the condition of the lying area and access to pasture were linked to the prevalence of lameness, lesions or swellings, and dirty hindquarters. While no common factors were found for displacements and lameness, lesions/swellings, and dirty hindquarters, these indicators were primarily influenced by the quality of walking and lying surfaces. The frequency of displacements was associated with factors linked to limited resources (86).

By utilizing measures like “Body condition score” and “Cleanliness of observed body parts,” the evaluation of dairy cows’ well-being in permanent tie-stalls versus those with pasture access has effectively emphasized the significance of high-quality housing (87). This, in turn, enhances animal performance, impacting their health and productivity positively. The assessment of QBA has underscored the value of granting animals freedom and the opportunity for unrestricted movement, enabling the natural display of physiological behaviors (87).

A pilot study from 2017 aimed to compare the welfare of dairy cows in tie-stall (TS) and open-stall (OS) systems (88). Various health and stress-related parameters were measured in 80 lactating cows across eight farms. The study found that the housing system influenced certain indicators like ALT (alanine- aminotransferase), BHBA (β-hydroxybutyrate), OFR (oxygen free radicals), and cortisol levels, with OS showing higher OFR potentially due to increased productivity demands. Overall, while some parameters were affected, no significant signs of suffering were observed in either system, leading to the conclusion that the tie-stall system did not display notable welfare issues when compared to open-stall (88). In another study on the same topic, metabolic, immunological, and stress-related parameters in 155 cows across 18 farms in Tuscany were analyzed (89). Results revealed that the housing system influenced several parameters, with oxygen free radicals (OFR) levels higher in the OS system, likely due to increased productivity. Cortisol levels did not suggest chronic stress. The study concluded that, based on physiological parameters, cows in the TS system showed no severe signs of impairment. Notably, parameters like lysozyme (SL) and OFR had more favorable values in the TS group compared to OS, and no evident distress signs were observed in either group (89).

Two traditional farming systems (semi-intensive and intensive) in Sicily were also examined (90). Using a multicriteria system based on the European Food Safety Authority (EFSA) model, 18 dairy farms were assessed for welfare and health measures. Overall, the study concluded that the semi-intensive approach in Sicily better meets animal welfare conditions compared to the intensive system (90).

Improperly designed cubicles can lead to skin problems, lameness, and dirtiness (91). While recommendations from the International Commission of Agricultural and Biosystems Engineering exist, their effectiveness varies. The paper of Lardy et al. aims to enhance these recommendations by analyzing cubicle features and their relation to cow welfare indicators across 76 farms with 2,404 cows. The results highlight key factors such as obstacle placement, bedding material, and cubicle dimensions that impact cow welfare (91).

### 3.7 Chewing muscle activity

The sensor system designed to quantitatively and qualitatively assess chewing muscle activity in dairy cows, is called DairyCheck (92). It employs Electromyography (EMG) principles, with skin-affixed electrodes gaging potential shifts during chewing muscle contractions (masseter). This facilitates personalized quantitative and qualitative evaluations, forming a basis for early ailment detection. Results demonstrate minimal variations in individual chewing phases. Daily chewing spans around 7 h, comprising roughly 15 phases each lasting about 28 min. Notably, nocturnal chewing variances are less pronounced, potentially aiding the detection of significant behavioral shifts during the night. The DairyCheck demonstration underscores its capacity to distinguish chewing from other oral actions. Further exploration aims to characterize eating-related oral activities and distinguish “other activities,” with the goal of deducing feeding behavior from muscle activity (92).

### 3.8 Heart rate

As sentient beings, dairy cows experience a range of emotions and physical responses to their environment and overall health. Monitoring the heart rate of dairy cows as a tool for dairy farmers and researchers to assess their welfare has been the subject of numerous studies. This non-invasive and real-time measurement offers insights into various aspects of a cow’s life, including its response to stress, pain, and environmental conditions.

Data was collected from 219 Holstein cows in different types of farms to study the impact of posture, rumination, and feeding on heart rate (HR) and heart rate variability (HRV) (93). The study found that sympathetic activity increased in the following order: when cows were lying, ruminating while lying, standing, ruminating while standing, and feeding. The vagal activity decreased in the same order in both smaller and larger-scale farms. The study also found that cows in larger-scale farms had lower vagal activity but higher sympathetic activity compared to cows in smaller-scale farms, suggesting potential welfare concerns related to social stress (93).

Erdmann et al. (94) aimed to investigate whether HRV parameters could serve as early indicators of metabolic stress in high-performing dairy cows. The researchers focused on evaluating the autonomic regulation and stress levels of 10 pregnant dried-off German Holstein cows throughout a 10-h fasting period, examining their conditions before, during, and after. They found that by examining HRV frequency domain parameters, cows could be retrospectively grouped based on their response to food removal, with some showing increased parasympathetic activity and others showing decreased activity. These findings suggest that HRV parameters could potentially be used as predictive markers for detecting alterations in autonomic regulation before metabolic disturbances occur (94).

A study was conducted on dairy cows milked in a high-capacity rotary milking system to assess their stress responses during the milking process (95). The researchers analyzed HR, HRV, rumination behavior, and step behavior during different stages of milking. The findings indicated that driving the cows to the holding pen caused an increase in HR and a decrease in vagal tone, while being in the holding pen resulted in decreased vagal tone and increased sympathetic tone. However, during milking, there was a recovery of autonomic activity, with increased vagal tone and decreased sympathetic tone, along with a low frequency of steps and a high prevalence of rumination, suggesting potential welfare benefits of the rotary milking system (95).

According to Hunter et al. (96), analyzing dairy cow sleep patterns is crucial for understanding their well-being amid environmental changes or other stressors. The current gold standard, polysomnography (PSG), can be challenging to conduct. In the study from 2021, HR and HRV were compared with PSG in two dairy cow groups, considering the impact of lying postures. Results showed HR decreasing with sleep depth, higher HRV during REM sleep, and lying postures influencing HR and HRV. Patterns were consistent across both groups, suggesting that HR and HRV changes correspond with sleep stages in cows. The findings also indicate associations between sleep stage, HR, and HRV, emphasizing their practical use in identifying sleep stages in dairy cows and enhancing accessibility for animal welfare research (96).

In another approach, sleep stages were monitored in 19 Swedish dairy cows during different lactation stages (97). Using electrophysiological recordings, REM and non-REM sleep, drowsing, awake, and rumination were examined. Results showed variations in REM sleep during lactation, with the shortest duration observed 2 weeks post-calving. Significant differences in REM sleep bouts were noted between various lactation stages. Nighttime predominantly hosted REM sleep and rumination. The study emphasizes the importance of considering lactation stage in future dairy cow sleep research (97).

Jurkovich et al. (98) compared HRV in dairy cows in a small-scale dairy farm in Hungary during traditional parlor milking and later automated milking. The purpose was to assess stress related to milking type and human interaction frequency. Parlor milking involved more frequent human contact and animal movement. The study found that automated milking appeared less stressful for cows, attributed to shorter post-milking restraint and reduced human interaction. The parameters measured included HRV, fecal glucocorticoid concentrations, and avoidance distance. The results suggest that automated milking may be less stressful for dairy cows, with potential implications for improving animal welfare in conventional milking systems (98).

In a literature review by Kovacs et al. (99), it is described that there are different studies that highlight HRV as a more precise indicator of autonomic nervous system activity in dairy cattle. Effective in detecting stress related to routine practices, pain, and milking, HR and HRV play a crucial role in understanding dairy cow welfare. Future research opportunities include evaluating milking as a stress source and exploring the impact of chronic stressors, emphasizing the need for ongoing studies to enhance our understanding and improve overall animal welfare (99).

In a study from 2013, HR and HRV during milking in a parallel milking parlor were investigated (100). The results showed that there was no significant difference in animal welfare between the reference period and the different phases of milking. However, HRV parameters were significantly affected by factors such as parity, breeding bull, and milk production. Primiparous cows were found to be more susceptible to the milking process compared to multiparous cows. Overall, the study suggests that the conventional milking process is not highly stressful for cows, but certain factors can influence their physiological response (100).

### 3.9 The role of biomarkers, milk parameters and cortisol in welfare assessments

According to a 2015 review, enhancements in animal productivity have compromised fitness, leading to increased susceptibility to diseases and reproductive issues (101). Future breeding strategies should aim to strike a balance between high production and health, relying on both validated and new biomarkers for insights into physiological aspects. These biomarkers play a crucial role in comprehending adaptation to diverse environments, thereby contributing to welfare assessment and refining management and breeding practices. Subsequent studies should focus on identifying welfare biomarkers, developing cost-effective monitoring techniques, and exploring variations among bovine dairy breeds. Automated technologies hold the promise of precise quantification of animal responses, while biomarkers of robustness guide breeding for resilient animals (101).

In another review titled ‘Engineering to Support Wellbeing,’ it is noted that current EU livestock policies prioritize the well-being of dairy animals, addressing challenges such as health issues and fertility conflicts (102). Despite technological advances, the productive lifespan of dairy cows is limited, emphasizing the complexity of their management. Assessing dairy animal welfare involves both objective and subjective measures. The presented DairyCare project aims to enhance well-being through technological advances, integrating biomarker-based, activity-based, and systems-level welfare technologies. The livestock sector’s technological focus heavily relies on RFID devices for monitoring and managing cows. Precision Livestock Farming (PLF) integrates RFID, IoT (Internet of Things), and SNO (smart networked objects) to monitor animals for optimal production. PLF provides opportunities for enhancing animal well-being, with wearables like accelerometers and automated milking systems contributing to data-driven decision-making in livestock management (102).

A review by Zachut et al. (103) underscores recent endeavors in identifying fitness, stress, and welfare biomarkers in dairy cows, particularly markers linked to energy balance, oxidative stress, and production-related diseases. The paper also highlights the necessity for future research and technological advancements, specifically in integrating established biomarkers into automated systems for practical use by farmers and veterinarians. Collaborative efforts across diverse disciplines and the adoption of PLF are crucial for improving dairy animal performance, health, and welfare (103).

The following systematic review from 2021 aimed to review PLF technologies for real-time welfare assessment in dairy cattle (104). Out of 1,111 publications, only 42 studies on 30 tools met validation requirements. A market search identified 129 retailed technologies, but only 18 (14%) were externally validated. Accelerometers had the highest validation rate (30%), while cameras, load cells, milk sensors, and boluses had lower rates (7–10%). Validated traits included activity, feeding, physical condition, and health. Most tools were validated on adult cows, with non-active behaviors validated more frequently than active ones. According to the authors, PLF technologies currently have limited potential for assessing appropriate behavior in dairy cows, necessitating further validation studies, particularly in commercial herds, to enhance trust and applicability. Future research should focus on developing and validating PLF technologies for assessing appropriate behavior, as well as monitoring health and welfare in calves and heifers (104).

PLF technology was used to monitor variables like activity and vocalization in another approach (105). A study at a Dutch dairy farm aimed to correlate cattle vocalization with behavior, finding significant frequency differences during lying and ruminating. Adult dairy cattle had lower vocalization frequencies than heifers. Despite concerns about housing conditions affecting welfare, sound analysis showed potential as a dairy cattle management tool. The study recommended future research with better camera coverage and consideration of breed-specific vocalization variations (105).

In a comprehensive investigation, another study sought to unveil the reliability of milk yield as an insightful indicator of the welfare within dairy herds (106). Favorable connections emerged, linking milk production to reduced aggression among cows and a positive emotional atmosphere within the herd. The study encompassed 125 French dairy farms. However, a contrasting relationship was observed concerning good health, evidenced by instances of diseases and injuries. The interplay of these opposing factors yielded no conclusive correlation between milk production and the overall well-being of the herd. The research implies that, although adverse emotional experiences and suboptimal emotional states can adversely affect milk output, relying solely on milk yield is insufficient for gauging the comprehensive welfare of the herd, given its intricate interrelation with health issues (106). In a different approach, the experts came to the conclusion that collected bulk tank milk data might not be a reliable pre-screening tool for estimating dairy cattle welfare at the herd level due to very weak associations (107). Weak but statistically significant correlations were found between bulk tank milk parameters (somatic cell count, total bacteria count, urea, proteins) and welfare scores. These correlations were influenced by factors like dilution of individual cow milk and inclusion of non-lactating animals in welfare assessments. Despite the known link between milk parameters and udder health, correlations with overall animal welfare scores were weak. Total bacteria count showed partial confirmation of a link between farmer practices and animal welfare. Urea content displayed weak positive correlations with welfare scores, while no significant associations were found between fat content and welfare scores (107).

Jerram et al. (108) investigated stress levels in dairy cows during a transition from conventional milking to an automatic milking system (AMS). Stress, measured through cortisol levels in saliva and hair, can impact immunity and reproduction. AMS, associated with higher milking frequency and yields, showed varying effects. Non-lame cows exhibited reduced salivary cortisol levels post-AMS, while lameness and pregnancy affected salivary, not hair, cortisol. Hair cortisol increased after installation, possibly seasonally. AMS improved production, udder health, and milk yield, with no overall increase in cow stress (108).

In a paper from 2020, the physiological stress levels in dairy cows on 25 German organic farms were investigated, assessing cortisol metabolite concentrations in feces (109). The results showed decreased cortisol metabolite levels on farms that did not separate diseased cows, possibly indicating reduced regrouping stress. Lower levels were also observed on farms with straw yards and generous lying space. Increased human-animal contact was associated with decreased cortisol metabolite levels. However, unexpected results, such as higher levels on farms that fed concentrates by hand, suggest the complex and multifaceted nature of stress physiology in on-farm conditions. Overall, the study highlighted the importance of factors like resting comfort, human-animal contact, and feeding practices in influencing physiological stress levels in dairy cows (109).

To further investigate the correlation between cortisol concentrations in blood serum (KoB) and other non-invasive measures like saliva (KoS), tears (KoT), milk (KoM), and feces (KoK) in cows, Heinrich et al. (110) subjected cows to sham foot trimming (sKB) as an acute stress model. KoB and KoT increased during sKB, reaching a maximum at 60 min, while KoK peaked at 660 min. Significant correlations were found between KoB and KoT, KoK and KoB, and a trend for KoK and KoT during sKB. KoB significantly decreased from day 1 to day 4, then increased on day 5. KoS and KoT served as reliable proxies for KoB, while KoM exhibited differences. The study suggests non-invasive methods like tear and saliva collection can effectively measure cortisol, emphasizing the importance of calm cow handling for better welfare (110).

A study from 2021 aimed to compare eight welfare assessment protocols in relation to hair cortisol concentrations (111). Despite expectations, most protocols did not significantly correlate with hair cortisol levels, challenging the assumption that hair cortisol is a reliable indicator of cow welfare. The inconsistent correlation among protocols and their poor alignment suggests the need for further research to assess and potentially modify existing welfare assessment tools for accurate measurement (111).

The next approach aimed to pinpoint reliable indicators for assessing the well-being of dairy cows (112). The Animal Welfare and Biosecurity Evaluation form (AWB-EF), endorsed by the Italian National Center of Reference for Animal Welfare, was employed to evaluate 16 Sardinian dairy farms. Analyzing hematological parameters in 230 Holstein dairy cattle revealed a robust correlation between AWB-EF and laboratory findings. The study suggests that veterinarians can use a validated checklist alongside specific laboratory parameters to detect early signs of stress. It is noted that it is crucial to emphasize that evaluating animal welfare requires a multidisciplinary approach, and health assessment alone falls short of determining overall well-being (112).

### 3.10 Animal needs index

According to the substance of the following approach, the Animal Needs Index (ANI) assesses five aspects of the animal environment, including mobility, social interaction, flooring conditions, stable climate, and human care (113). They compared two organic and two conventional farms. Locomotion disorders, the first ANI category, were absent in the observed farms. The scoring considered tethered and free housing, with Farm No. 1 performing the best (85.71%) and Farm No. 3 the worst (69.2%). Cleanliness of the resting area, a part of ANI Category I, was a notable shortcoming on the farms, particularly on Farm No. 2. Social behavior, part of ANI Category II, showed unsuitable manifestations on Farm No. 3 due to tethering and lack of stable hierarchy. Bioclimate, addressing temperature and humidity, was assessed in ANI Category IV, with Farm No. 2 having the worst results. The fifth ANI category evaluated man-animal interactions and animal care, highlighting issues in farms with tethered animals (No. 2 and No. 3). ANI proved practical for welfare assessment, offering a rapid and repeatable method, but it was suggested that additional animal parameters be considered for a more comprehensive evaluation (113).

Hristov et al. (114) investigated the correlation between rearing systems, Animal Needs Index (ANI), and milk traits in five dairy farms. The rearing systems varied, with open stalls in farms A and C practicing loose cow rearing, while others tied cows in closed stalls. Outdoor pens were available in two farms. The total ANI scores ranged from A 35.5 to E 10.5, with farm A having excellent welfare levels. The rearing system significantly influenced cow welfare (*p* < 0.001) and had a notable impact on average daily milk yield, milk fat, and protein yield (*p* < 0.01). The study emphasized the importance of improving housing conditions based on ANI scores to enhance cattle production performance (114).

The authors of a paper from 2011 assessed the welfare of dairy cows in Romanian tie-stall and free-stall farms using the Austrian ANI 35 L/2000-cattle system (115). Among 40 cattle houses, free-stall barns demonstrated higher overall ANI scores compared to tie-stall barns. Welfare factors such as locomotion, social interactions, flooring, light, and air, along with stockmanship, consistently scored lower in tie-stall barns. The findings suggest that dairy cows experience better welfare in free-stall housing, highlighting the need for improvements in tie-stall barns (115).

### 3.11 Routine herd data and register data

In the invited review by de Vries et al. (116), the exploration of variables in routine herd data (VRHD) associated with dairy cattle welfare indicators (WI) is a key focus. Among the 27 VRHD and 34 WI under consideration, extensive associations emerged from 146 studies. Twenty-three VRHD demonstrated links to 16 WI, with particularly noteworthy connections to milk yield, culling, and reproduction. However, limited associations were noted for WI related to behavioral aspects, disease symptoms, or resources-based indicators (116).

Another paper on this topic investigated using routine herd data (RHD) from national databases (117). Trained observers collected welfare data for 41 indicators in Dutch dairy herds, while RHD were extracted from national databases. RHD served as predictors for various welfare indicators, showing high accuracy for some. Best-performing models included indicators like access to drinkers, percentage of very lean cows, cows lying outside the supposed lying area, and cows with vulvar discharge. RHD can serve as a prescreening tool to detect herds with welfare problems, but the predictive models require validation in additional field studies (117). The same authors published a study where they used RHD and housing and management (HM) data to estimate dairy herd welfare levels more efficiently (118). The observers collected welfare data for six indicators in Dutch dairy herds, while RHD and HM data were obtained. Predictions were moderately accurate for various welfare indicators, showing potential for screening herds efficiently (118).

Register data from Nordic dairy herds, widely available for research, were assessed for their utility in identifying herds with good welfare and distinguishing between those with deficiencies (119). On-farm animal-based measurements in 55 herds formed the basis for welfare classification. A case herd with “good welfare” had no scores lying among the 10% worst in any of nine welfare measurements, with 28 herds meeting this criterion. Subsequently, 65 potential welfare indicators from a national dairy database were identified. The final set, including fertility measures, cow mortality, stillbirth rate, mastitis incidence, and feed-related diseases, showed a high sensitivity (96%) but lower specificity (56%). Combining models significantly improved welfare classification, demonstrating the use of pre-collected register data for approving dairy farms with good welfare and enhancing herd welfare assessment (119).

A study from Denmark aimed to assess register data variables as predictors of dairy herds violating animal welfare legislation (VoAWL) (120). VoAWL includes the presence of injured animals not separated or those warranting euthanasia still in the herd. Analysis of 73 Danish dairy herds identified predictors: increasing milk yield variation in first lactation cows, high bulk tank somatic cell count (≥250,000 cells/ml), and a suspiciously low number of veterinary treatments (≤25 treatments/100 cow years). These predictors suggest underlying management issues affecting animal welfare. Further investigations are required for causal inferences, emphasizing the need for comprehensive risk factors beyond legislative standards (120).

### 3.12 Cleanliness

Another study investigated the influence of cleanliness on cattle health, welfare, and farm profitability (121). In Sweden, despite legislation requiring animals to be ‘clean enough,’ official inspections find a significant prevalence of dirty cattle. Among 371 inspected farms, 49% had dirty cattle, but not all were considered non-compliant. The study highlights management routines as a key factor affecting cattle cleanliness. Farmers with clean cattle prioritize access to bedding material, while those with dirty cattle suggest shorter slaughter queues as a remedy. The research emphasizes the necessity for clearer guidelines in determining compliance with animal welfare legislation regarding cattle cleanliness (121).

In 2017, an Austrian dairy company introduced a third-party animal-based assessment to drive welfare improvements on farms (122). Analyzing data from 1,221 farms and 23,749 cows, prevalent welfare issues included dirty hind legs, signs of diarrhea, and hairless patches on the tarsal joint. Severe problems like very lean cows were rare. Generalized linear models revealed associations between milk delivery per cow, housing system, assessment period, and welfare outcomes. Some characteristics, however, had both positive and negative impacts, emphasizing the need for careful management to avoid undesired effects (122).

### 3.13 Reproduction

The effects of low and high concentrate supplementation on welfare, health, and reproduction in two dairy cow breeds on mountain farms were investigated in a study from Italy (123).

Contrary to expectations, higher concentrate levels did not necessarily result in lower animal welfare in alpine regions. One breed showed benefits with a lower calving interval and more lactations. However, caution in interpreting results is advised due to noted weaknesses in group comparison (123).

In another approach, the aim was to assess the impact of oestrus intensity and alternative indicators, such as progesterone recordings, on the reproductive performance of dairy cows (124). Results showed that heifers had a higher pregnancy rate than first-parity cows, and standing oestrus significantly increased the odds of pregnancy and calving. The eProCheck800 ELISA reader, monitoring progesterone, complemented on-farm reproductive management but had less accuracy than visual oestrus detection. Oestrus intensity was linked to good welfare, evidenced by higher pregnancy rates, emphasizing the importance of optimal oestrus expression in high-producing dairy cattle (124).

### 3.14 Post mortem

Knock and Carroll explored using abattoir meat inspection data to assess cattle welfare (125). They examined associations between ante-mortem issues like lameness and body condition with post-mortem measures. Results suggest recording carcass weight and bruising during meat inspection as indicators of welfare. Associations between ante-mortem indicators and post-mortem measures vary by cattle characteristics. The prevalence of bruises underscores their importance in welfare assessments. The findings propose post-mortem measures as potential indicators of cattle welfare, urging further research to establish on-farm welfare associations (125).

Another paper on this topic presents an innovative approach to retrospectively assess cattle welfare at the abattoir using claw disorders (126). The findings, based on the analysis of 1,040 cattle from various production systems, reveal a high prevalence of abnormal claw shapes and claw wall fissures. Notably, associations between lesions in front and rear limbs varied by production system. Feedlot and free-range cattle with white line disease and skin wounds showed higher meat pH. Claw disorders serve as valuable indicators of animal fitness, reflecting their ability to cope with husbandry and pre-slaughter conditions. The importance of retrospective abattoir-level claw assessment as a tool to understand how production systems impact cattle health and welfare is pointed out. It is noted that these measures, treated as iceberg indicators, can be integrated into protocols for post-mortem cattle welfare assessment (126).

### 3.15 Eye white, ear posture and nasal temperature to understand cows emotions

Battini et al. (127) explored using eye white and ear posture as indicators of emotions in dairy cows. The research on five Italian dairy farms, analyzed 436 cow head photos, revealing strong correlations. Contexts like pasture access and human-animal interaction impact emotions. The study emphasizes the feasibility of on-farm assessment using photos and concludes that eye white and ear posture are valuable indicators for evaluating dairy cows’ emotional well-being (127).

Another paper about ear postures as indicators of positive, low-arousal emotional states in dairy cows: Through 381 focal observations on 13 cows, four ear postures (EP1 to EP4) were analyzed during baseline, stimulus (stroking), and post-stimulus segments (128). The findings suggest that EP1 and EP2, considered relaxed postures, were more prevalent before and after stroking, while EP3 and EP4, associated with arousal, increased during stroking. These results propose that relaxed ear postures may signify positive emotional states in dairy cows. The study suggests that ear postures could serve as both immediate indicators and reflections of longer-lasting mood states in cows (128).

The use of visible eye whites as an indicator of positive emotional states in dairy cows during stroking was also investigated (129). While not currently suitable for on-farm use due to analysis time, the measure holds potential for research on emotional arousal. Further studies are needed to explore its applicability in different contexts and species (129).

A further study by Proctor et al. (130) focused on whether positive emotions affect nasal temperatures in cows. Through 350 focal observations, they induced positive emotional states in cows by stroking them. The results showed a significant decrease in nasal temperature during stroking, suggesting a change in valence. This challenges the notion that emotional fever is only associated with negative states. While nasal temperature may be a useful measure of emotional state, further research is needed (130).

### 3.16 Other approaches

A study from 2018 investigated the use of outcome-based observations in Assured Dairy Farm (ADF), Soil Association Organic Standards (SA), and cross compliance (CC) farm assessment reports (131). ADF reports had a higher response rate (61.0%) with resource-based comments, while SA and CC reports showed significantly more outcome-based comments. ADF comments were mainly compliant and resource-based, serving as proof of assessment. SA, emphasizing welfare outcome measures, increased outcome-based comments. CC prioritized outcome-based evidence for noncompliant decisions. The study suggests the need for a balance between general and detailed comments and proposes in-depth interviews for exploring individual rationale in future assessments (131).

In a study analyzing inspections in Swedish dairy herds from 2010 to 2013, conducted separately by the County Administrative Board (CAB) and Arla Foods, common non-compliances were identified (132). Dirty dairy cattle was a frequent issue in both systems, but substantial differences suggested distinct focuses. Risk factors for non-compliance included tie-stall housing, winter season, and, notably, overall organic farms demonstrated fewer predicted non-compliances than conventional ones (132).

An investigation by Mattiello et al. (133) aimed to compare welfare indicators among five Italian cattle breeds (Italian Holstein-Friesian, Italian Bruna, Pezzata Rossa Italiana, Grigia Alpina, and Pezzata Rossa d’Oropa) kept in tie-stalls in the Italian Alps. The study assessed integument alterations, lameness, and physical malformations in 612 cows. Results revealed a decreasing trend in welfare problems from more to less productive breeds, with local breeds exhibiting lower prevalence. Italian Holstein-Friesian generally showed the highest percentage of issues. Housing in tie-stalls was associated with welfare concerns, emphasizing the need for genetic selection changes in the dairy industry (133).

A protocol, developed for integrating herd welfare assessment into Dutch dairy farming’s quality assurance program, was tested in a pilot study involving 52 herds (134). The final protocol, consisting of 16 animal-based and 14 environment-based parameters, was utilized in a voluntary field survey of 164 herds, with an average assessment time of 78 min per herd. The protocol aimed at periodic welfare auditing, emphasizing cows’ biological needs. Focused on cow behavior for feasibility, the final protocol received widespread agreement among stakeholders (134).

A survey on dairy cow welfare in 7 Italian regions involved 943 farms (135). Using a checklist with 303 parameters, categorized into direct and indirect criteria covering farm management, housing, environment, feeding, and health, the study assessed animal welfare. Parameters were evaluated based on legislation and a semi-quantitative scale. Among the 249 examined, 15 had a failure prevalence below 1%, while non-compliance prevalence ranged from 2 to 67%, inversely proportional to herd size. Common non-compliance aspects related to calves management, staff training, and prophylaxis programs. Larger farms exhibited lower non-compliance, highlighting the importance of technology and staff training for better herd health. The combination of direct and indirect criteria aligns with EU animal welfare recommendations (135).

To enhance animal welfare, the Italian National Reference Center for Animal Welfare (CReNBA) promotes 38 best practices for dairy cattle (136). Covering managerial and equipment factors, these practices shift towards “positive animal welfare” (PAW), considering a life worth living. CReNBA’s welfare assessment protocol, part of the “ClassyFarm” system, incorporates hazards and benefits for a comprehensive guide (136).

A study by Pezzuolo et al. (137) introduces a cost-effective 3D camera system for frequent growth assessment of calves and cows. Verified for accuracy through uncertainty analysis and calibration, the system showed generally precise measurements, with deviations under 6% compared to manual measurements, except for specific parameters. With increasing stock densities on dairy farms, the non-contact measurement approach becomes valuable (137).

In a survey involving 16 Italian veterinarians, a Delphi technique was used to assess hazards and welfare promoters in loose housing systems for dairy cows (138). Hazards affecting lactating cows, such as inadequate flooring and lack of bedding, were rated high. Welfare promoters, including optimal resting conditions and skilled stockpersons, received top ratings. Animal-based measures like lameness observation and mortality records were considered crucial (138).

A paper by Katzenberger et al. (139) assessed the feasibility of farmers’ self-assessment for a dairy cattle welfare assurance program in South Tyrol. The inter-rater reliability between experts and farmers in assessing welfare outcomes was found to be slight to moderate (139).

## 4 Discussion/conclusion

This comprehensive systematic review accentuates the pivotal role of the WQ® protocol in evaluating dairy cattle welfare, acknowledging its versatility in identifying risk factors and assessing various parameters. Despite its acknowledged effectiveness, challenges like time consumption persist, prompting ongoing innovative efforts for protocol refinement and alternative assessment methods. Benchmarking, exemplified in diverse welfare assessment protocols, serves as an important tool for targeted improvements and overall welfare enhancement. Correlations between lameness, mobility scores, and adverse effects on production underscore the need for early identification through technology. Dairy cow behavior analysis provides valuable insights into their well-being, emphasizing the importance of understanding and enhancing welfare through various measures.

Exploring the human-animal relationship in dairy farming is pivotal for ethical considerations and welfare choices. Housing and pasture systems significantly impact dairy cow welfare and productivity, with studies favoring pasture-based systems. The DairyCheck sensor system, monitoring chewing muscle activity, showcases promising capabilities for personalized evaluations and early ailment detection. Heart rate and heart rate variability monitoring offer valuable insights into welfare, with automated milking systems presenting potential advantages. Biomarkers play an essential role in balancing productivity and health, as shown in the DairyCare project. Cortisol is a promising biomarker for assessing dairy cow welfare due to its ability to effectively reflect stress levels. It can be measured non-invasively in methods such as saliva, tears, and feces, minimizing stress on the animals during sampling. Further research should continue in this direction to enhance understanding and application.

Precision Livestock Farming offers real-time welfare assessment, but validation is important. Monitoring vocalization, correlating milk yield with well-being, and assessing bulk tank milk data reveal complex relationships between productivity, emotional experiences, and overall welfare. Stress physiology is multifaceted, influenced by factors like resting comfort, human-animal contact, and feeding practices. A multidisciplinary approach provides a comprehensive understanding of early signs of stress and contributes to overall well-being assessment in dairy cows.

The Animal Needs Index offers a rapid method for assessing dairy cow welfare, emphasizing the influence of rearing systems. Routine herd data analysis reveals significant links with milk yield, culling, and reproduction, aiding in prescreening for potential welfare concerns. Predictors of dairy herds violating animal welfare legislation underscore the importance of comprehensive risk factors. Cleanliness emerges as a relevant factor in cattle management, impacting health, welfare, and farm profitability. Unexpected outcomes in concentrate supplementation caution against simplistic interpretations, while optimal oestrus expression proves vital for reproductive performance.

Diverse approaches, including abattoir data analysis, claw disorders, visual indicators of emotions, and innovative technologies, contribute valuable insights into cattle welfare assessment. The integration of outcome-based observations, breed-specific considerations, and the development of practical protocols and technologies further advance our understanding and ability to enhance dairy cattle welfare across various farming systems.

Commercial animal welfare audits must rely either on easily observable well-being indicators or on information from herd records. The ability to measure biomarkers or heart rate variability during an audit is limited due to several practical and logistical reasons. Measuring biomarkers and monitoring heart rate variability require specialized equipment and expertise, which are often expensive and not easily portable for use during an audit. Additionally, the analysis and interpretation of the results require time and expertise, which may not always be available during a standard animal welfare audit on a farm. Efficiency and time-effectiveness are important for animal welfare assessments, especially considering the limited time available for audits and the potential slowdown caused by complex measurement methods.

For these reasons, easily observable well-being indicators such as BCS, lameness, claw health, cleanliness, and somatic cell count provide practical and readily accessible data. These can be assessed without special equipment, making them ideal for use during an audit. Additionally, information from herd records can offer valuable insights into animal well-being, including feed rations, health treatments, reproductive data, and milk production. These data are often well-documented and easily accessible, facilitating their integration into animal welfare audits.

The information gained from this systematic review can seamlessly integrate into existing commercial animal welfare assessments. Indicators such as BCS, lameness, claw health, and cleanliness offer practical and measurable criteria that can be easily incorporated into routine assessments. This allows farmers and auditors to promptly respond to potential issues and take targeted actions to improve animal well-being.

Further research into abbreviated protocols, such as the DCF protocol, would be beneficial. The DCF protocol showed correlations with the WQ® protocol while requiring significantly less time, suggesting that streamlined approaches could offer practical alternatives without compromising assessment quality. It saves approximately 6 h on a farm with 200 dairy cows, making it much more feasible for regular assessments. This is more beneficial for farmers balancing numerous daily tasks, ensuring that welfare evaluations can be integrated into routine operations without major disruptions.

The DCF protocol relies on simpler, more direct indicators that are quicker and easier to assess, such as BCS, lameness, and cleanliness. These animal-based measures provide immediate feedback and are practical to evaluate during routine checks. In contrast, the WQ® protocol includes more complex evaluations that can be time-consuming and require specialized training.

Additionally, the DCF protocol uses a more transparent and straightforward method of summarizing welfare measures. Unlike the WQ® protocol’s complex weighting and aggregation methods, the DCF protocol’s summarization is easier to understand and implement, ensuring that farmers can readily interpret and act on the results.

Furthermore, it requires inspecting fewer cows (16% of the population) compared to the WQ® protocol (38%), contributing to its time efficiency and practicality without significantly compromising accuracy.

In summary, the design of the DCF protocol makes it a more suitable tool for everyday use by farmers and in commercial animal welfare audits. Given these promising results, further studies should be conducted in this direction to gather more data on the DCF protocol. This would enable the direct use of the DCF protocol itself or the development of a similar standardized protocol that is comparably accurate to the WQ® protocol. Establishing a protocol from existing validated indicators that, when combined, offer a comprehensive and objective overview of the welfare status of cows would facilitate standardized and easily comparable assessments of animal welfare.

## Data availability statement

The original contributions presented in the study are included in the article/supplementary material, further inquiries can be directed to the corresponding author/s.

## Author contributions

JL: Writing – original draft, Data curation, Formal analysis, Investigation, Methodology, Visualization. CT-R: Writing – review & editing, Conceptualization, Methodology, Project administration, Supervision. RM: Writing – review & editing, Conceptualization, Methodology, Project administration, Supervision.
